# The Characterisation of Diarrhoeagenic Verotoxin Producing Non-O157 Escherichia coli among Young Children in Kuantan, Malaysia

**DOI:** 10.21315/mjms2022.29.2.6

**Published:** 2022-04-21

**Authors:** Md Fazlul KARIM KHAN, Shah Samiur RASHID, Aizi Nor Mazila RAMLI, Muhammad Nomani KABIR, Mohammad Nazmul HASAN MAZIZ

**Affiliations:** 1 Faculty of Industrial Sciences Technology, Universiti Malaysia Pahang, Pahang, Malaysia; 2 Department of Computer Science and Engineering, Trust University Barishal, Barishal, Bangladesh; 3 Faculty of Medicine, Bioscience and Nursing, MAHSA University, Selangor, Malaysia

**Keywords:** children, diarrhoea, Escherichia coli, non-O157 E. coli, Malaysia

## Abstract

**Background:**

Diarrhoeagenic verotoxin producing non-O157 *Escherichia coli* (VTEC) are associated with endemic infantile diarrhoea-causing morbidity and mortality worldwide. VTEC can also cause severe illness and has an impact on outbreaks, especially in developing countries. This study aims to investigate the prevalence and characterisation of VTEC and their association in causing infectious diarrhoea among Malaysian children.

**Methods:**

Standard microbiological techniques identified a total of 137 non-repeated, clinically significant *E. coli* isolates. Serological assays discerned non-O157 *E. coli* serogroup, subjected to virulence screen (VT1 and VT2) by a polymerase chain reaction (PCR).

**Results:**

Different PCR sets characterised the 49 clinical isolates of sorbitol positive non-O157 *E. coli*. Twenty-nine isolates harboured verotoxin genes associated with diarrhoea among children (≤ 5 years old). Among the 29 (59.18%) strains of verotoxin producing *E. coli*, genotypes VT1 and VT2 were detected in 21 (42.85%) and 5 (10.20%) isolates respectively, while both VT1 and VT2 genes were confirmed in 3 (6.12%) isolates.

**Conclusion:**

This study evaluates on the prevalence, serological characteristics and antimicrobial susceptibility patterns of VTEC diarrhoea affected children (≤ 5 years old). Besides, the prevalence of verotoxin gene was determined as a root cause of diarrhoea among Malaysian children.

## Introduction

Diarrhoea is one of the leading causes of children (≤ 5 years old of age) morbidity and mortality in developing countries (1) while the second leading cause of death globally (2). The diarrhoeagenic *Escherichia coli* (*E. coli*) virulent factors cause diarrhoea due to six major pathotypes (1). In Malaysia, acute diarrhoea is still a significant public health concern (3).

Verotoxin producing non-O157 *E. coli* (VTEC) is progressively well-known as an essential enteric foodborne pathogen. VTEC is one of the most frequent causes of diarrhoea. However, the potentially life-threating complications of VTEC infections is highlighted as a public health problem (4). The production of verotoxin related virulence properties causes mild diarrhoea, haemorrhagic colitis (HC) and fatal haemolytic uremic syndrome (HUS) across the worldwide (5). Generally, *E. coli* O157: H7 serotypes associated with foodborne illness and non-O157 serotypes have been accused of gastroenteritis and HUS outbreaks (5). Furthermore, VTEC allied with severe foodborne illness, resulting in bloody diarrhoea with hemorrhagic colitis and the hemolytic uremic syndrome (6–7). To date, no outbreaks were reported due to VTEC in Malaysia (8). The pathogenic virulence properties of some VTEC serogroups; O26, O121, O103, O111, O145 and O45, signified as the ‘big 6’ VTEC serogroups (9–10). However, a total of 380 different serotypes of VTEC strains frequently associated with human infections (11). In recent times, several outbreaks occurred due to VTEC serotypes, including O26: H11, O103: H2, O104: H4, O111: NM and O145: NM (12). VTEC O26 strains of non-O157 are the most prevalent cause allied with HUS and bloody diarrhoea in several European countries (5, 13).

The apparent rise in VTEC illness may be a consequence of improvements in testing for VTEC, which increases awareness among clinicians and diagnosticians. Recent studies estimated that 20%–60% of VTEC infections due to non-O157 strains were associated with numerous disease (14). Geographically in Europe, the disease caused by non-O157 serotypes is more prevalent compared to O157: H7 (5). In Malaysia, non-O157 *E. coli* exhibits significantly while the global incidence of VTEC infections exceeds O157 VTEC and the ratios between 2:1 to 7:1 have been reported (5, 8). VTEC serotypes are more prevalent compared to other virulence agents, and humans get easily infected by the virulence factors of VTEC through interaction with animals or consumption of contaminated meat, milk, vegetables, fruit and water by animal faeces (15). VTEC infection requires ingestion, with the bacterium potentially transmitted through contaminated food, water or person-to-person (16–18).

There is a scarcity of data on non-O157 *E. coli* serotype prevalence and virulence gene distribution, which is critical for the development of public health protection monitoring and control activities. The present study aimed to determine the prevalence of VTEC strains and to assess their virulence patterns as sources of infection among children ≤ 5 years old in Kuantan, Malaysia.

## Methods

### Bacterial Isolates

This study included 137 infants and young children (≤ 5 years old) infected by diarrhoea at Hospital Tengku Ampuan Afzan, Kuantan, Malaysia, from September 2018 to April 2019. This cross-sectional demographic information was obtained from patients, including age, sex, the onset of diarrhoea, antibiotic intake, relevant clinical and laboratory results. Children were enrolled if they had three or more liquid, semiliquid or bloody stools excretion per day. Most of the children with acute diarrhoea showed abdominal pain, followed by fever and vomiting. Our barring criteria were > 5 years old, no diarrhea, partial data, attributed to *Salmonella*, *Shigella*, or other types of bacteria and contaminated samples. Moreover, data collection was performed for comparison with these results.

### Microbiological Study

In this prospective study, 137 stool specimens were collected in clear, transparent, wide-mouthed sterile bottles and immediately transported to the microbiology laboratory. Consistency, colour, and mucous, blood and parasites of the specimens were examined. All the stool specimens were plated on MacConkey agar (Oxoid, Basingstoke, United Kingdom) and incubated (Thermo Scientific, USA) aerobically 24 h at 37 °C. Suspected colonies were plated on brain-heart infusion (BHI) agar (Oxoid, UK) and performed standard biochemical (oxidase, urease, citrate, indole and hydrogen sulfide production) assay to confirms suspected colonies as *E. coli* according to El-Hadedy and Abu El-Nour (19). All the biochemically confirmed *E. coli* isolates were additionally screened on cefixime tellurite sorbitol MacConkey agar (CT-SMAC) (Merck, Germany). *E. coli* O157: H7 serotypes appeared colorless (non-sorbitol fermenters), while non-O157: H7 seemed to be pink (sorbitol-fermenters) as described by previous researchers (20–23). Biochemically confirmed *E. coli* isolates were pre-enriched by BHI broth (Oxoid, UK) at a ratio of 1:10 and with 20% glycerol stored at −80 °C for further procedures.

### Serotyping Assays

All the biochemically identified *E. coli* isolates (lactose positive and negative) were selected for serotyping. Determination of non-O157 *E. coli* serogroups was performed using the O157 latex agglutination test kit (Oxoid, Basingstoke, UK) and Remel^TM^ slide agglutination test kit of polyvalent 2, 3 and 4 *E. coli* agglutinating sera (Thermo Scientific, USA) according to the manufacturer instructions. In the O157 latex agglutination test kit, isolates negative for agglutination were measured as non-O157 *E. coli* (24). For testing, a drop of polyvalent antisera was placed on a sterile slide. Each isolate was added to the antiserum. After 30 sec, samples were evaluated for agglutination. Moreover, agglutinated strains with polyvalent antisera were then tested with monovalent O antisera for the determination of non-O157 serotypes (O26, O121, O145, O103, O111 and O45).

### Determination of VT genes

*E. coli* isolates were inoculated in BHI broth (Oxoid, UK) at 37 °C aerobically and subjected to detect the presence of VT genes (VT1 and VT2) using polymerase chain reaction (PCR) protocol of Cebula et al. (25–26). DNA templates were prepared by the boiling method (27). Three to five colonies of *E. coli* were mixed in 50 μL of deionised water. The suspension was boiled for 10 min at 95 °C and centrifuged (Eppendorf, USA) for 10 min at 10,000 rpm. The formation of concentrated supernatant containing DNA was assessed by NanoDrop spectrophotometer (Thermo Scientific, USA). This obtained DNA template was subjected to PCR (Eppendorf Mastercycler gradient, USA). Determination of targeting virulence properties genes was examined using PCR with the specific primers and conditions (Table 1) as described previously (25–26). All the commercially manufactured oligonucleotide primers were obtained from Apical Scientific Sdn Bhd, Malaysia. The amplified DNA templates were separated by 1.5% agarose gel electrophoresis stained with 0.5 μg/mL GelRed (Biotium, USA) and examined for DNA under ultraviolet light using gel documentation system (Amersham Imager 680, USA). All the PCR products were purified by QIAquick PCR purification kit (QIAGEN, USA) according to the manufacture’s guidelines. After purification, the molecular weight of the DNA was determined and compared with the standard DNA molecular weight (1 kb DNA ladder) marker (QIAGEN, USA). This obtained purified DNA was sent for sequencing at Apical Scientific Sdn Bhd Malaysia. Sequences were analysed using the BLAST programme for the nucleotide database (https://blast.ncbi.nlm.nih.gov/Blast.cgi) and aligned with the sequence of the VT1 and VT2 gene.

### Antimicrobial Susceptibility Testing

The standard method (disk diffusion method) was performed for the determination of antimicrobial drug susceptibility, referring to the Clinical and Laboratory Standard Institute (CLSI) (29). Ten different types of antibiotic discs were tested: i) tazobactam/piperacillin (TZP)-10 μg/75 μg; ii) ceftazidime (CAZ)-30 μg; iii) gentamicin (GM)-10 μg; iv) ampicillin (AMP)-10 μg; v) imipenem (IPM)-10 μg; vi) cefuroxime (CXM)-30 μg; vii) cefotaxime (CTX)-30 μg; viii) ciprofloxacin (CIP)-5 μg; ix) amoxicillin-clavulanic acid (AMC)-30 μg and x) meropenem (MEM)-10 μg. Multi-drug resistance (MDR) was classified as acquired non-susceptibility to at least one (≥ 1) agent in three or more (≥ 3) antimicrobial categories (30). *E. coli* ATCC 25922 was used as quality control to determine susceptibility patterns (31). The CLSI guidelines were strictly followed for measurement of zone inhibition around the discs and interpretation of susceptibility patterns (sensitive, intermediate or resistant) of verotoxin producing *E. coli* (29).

### Statistical Analysis

Statistical analysis was obtained with Excel add-in Megastat, using the Pearson’s Chi-square test of independence and *P*-value (*P* ≤ 0.05) was considered significant.

## Results

**Figure 1:**
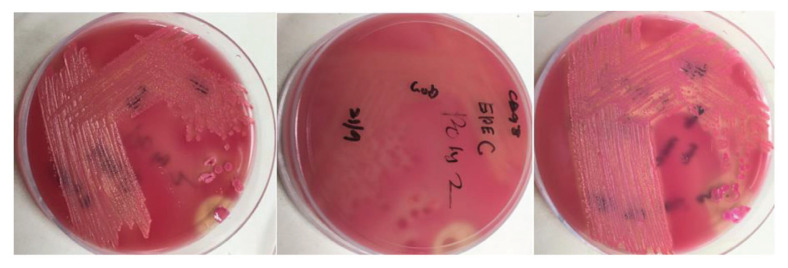
Formation of non-O157 *E. coli* on CT-SMAC culture plate media

A total presumptive 137 *E. coli* isolates collected from children (≤ 5 years old) were examined for virulence genes association with diarrhoea. Forty-nine isolates (35.5%) appeared pink (sorbitol-fermenters) on CT-SMAC media (Merck, Germany) after the overnight incubation (Thermo Scientific, USA) at 37 °C (Figure 1). These isolates were selected for further confirmation and characterisation.

Of the 137 diarrhoeic samples, 49 (35.5%) were *E. coli* non-O157 serogroups comprising O26 (*n* = 11 [8%]); O121 (*n* = 9 [6.5%]); O111 (*n* = 8 [5.8%]); O145 (*n* = 8 [5.8%]); O103 (*n* = 7 [5.1%]); and O45 (*n* = 6 [4.3%]) (Table 2). The presence of non-O157 *E. coli* isolates among children ≤ 5 years old were statistically significant (*P* < 0.001). Interestingly, children > 2 years old were highly infected with non-O157 (*n* = 38 [27.7 %]) *E. coli* compared to ≤ 2 years old (*n* = 11 [8 %]). Also, infection with non-O157 *E. coli* strains was observed to increase with age. Among the non-O157 *E. coli* serogroups, O26 was most frequently isolated (8%), followed by O121 (6.5%) and least O45 (4.3%). However, most of the non-O157 *E. coli* isolates were detected in watery stools (*n* = 25 [18.2%]), mucoid stools (*n* = 16 [11.6%]) and bloody stools (*n* = 8 [5.8%]), respectively.

Among the 137 children associated with diarrhoea, *n* = 17 (12.40%) children were 0 old months–5 old months of age followed by *n* = 36 (26.27%) children were 6 old months–12 months old, *n* = 22 (16.05%) were 13 old months–24 months old of age, *n* = 22 (16.05%) were 25 old months–36 months old of age, *n* = 25 (18.24%) were 37 old months–48 months old of age, and *n* = 15 (10.94%) were 49 old months–60 months old of age. The frequency of isolates among sex distribution was *n* = 71 (51.82%) male and *n* = 66 (48.18%) female, respectively. A total of 29 (21.16%) isolates were found to produce the verotoxin (VT1 and VT2) gene. A combination of VT1 and VT2 was found in 3 (2.18%) strains, while VT1 only was found in *n* = 21 (15.32%) isolates and VT2 only in *n* = 5 (3.64%) strains. Interestingly, the presence of the verotoxin gene has decreased with age. However, the most frequent, *n* = 8 (5.83%) VTEC isolates were detected in the age group of 13 old months–24 months old. There were significant differences in the frequency of the VTEC among the infants and children (males and females) aged ≤ 5 years old with respect to various clinical symptoms. The occurrence frequency significantly related to the presence of multiple features/risk factors (clinical symptoms, temperature, feeding types, admission, diarrhoea type and duration). However, the frequency of the VTEC was most prominent in females’ patients (65.51%). This effect is the most significant subsequent infection of higher severity and persists in all age groups. Similarly, the hospitalised patients (admitted for 4 days–5 days) with a higher temperature (62.06%) along with other clinical symptoms of nausea and vomiting (34.48%) and watery stools (48.27%) are relatedly significant in the higher incidence of pathogenic VTEC. Surprisingly, infants and children on breast milk are less infected compared to those who are on breast milk plus formula milk (51.72%). Besides, all the features of infections were relatedly significant (*P* < 0.005) risk factor for VTEC infections (Table 3).

**Figure 2:**
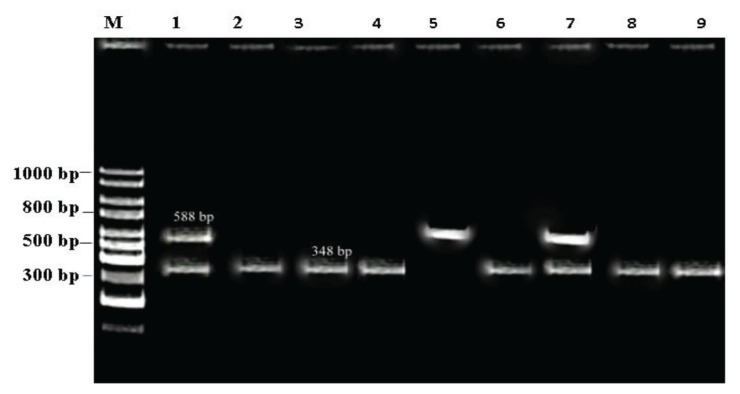
Confirmation of VTEC

All the 49 non-O157 *E. coli* were subjected to PCR using primers (VT1 and VT2). VT1 and VT2 gene were amplified, with amplicon sizes of 348 bp and 584 bp, respectively. A total of 29 (59.18%) isolates were found to produce the verotoxin (VT1 and VT2) gene. Twenty-one (42.85%) isolates were positive for the VT1 gene, followed by *n* = 5 (10.20%) VT2 gene, and *n* = 3 (6.12%) isolates were found to carry both VT1 and VT2 gene. Figure 2 represents the confirmed VT1 gene with amplicon sizes of 348 bp at lane 2, 3, 4, 6, 8 and 9 while VT2 gene of 584 bp were amplified at lane 5, respectively. At lane 7, both VT1 and VT2 gene were confirmed with amplicon sizes of 348 bp and 584 bp, respectively. For positive control *E. coli* O157: H7 was used at lane 1, which harbours both VT1 and VT2 gene while deionised water was used instead of template DNA as a negative control. PCR amplification specified that O26 and O45 were the leading serogroups carried verotoxin genes (18.36%; 9/49) and (10.20%; 5/49), respectively. A high proportion (*P* < 0.001) of the six serogroups non-O157 *E. coli* (O111, O121, O145, O26, O45 and O103) were significantly associated with variants genes (VT1, VT2 and, VT1 and VT2) (Table 4).

**Figure 3:**
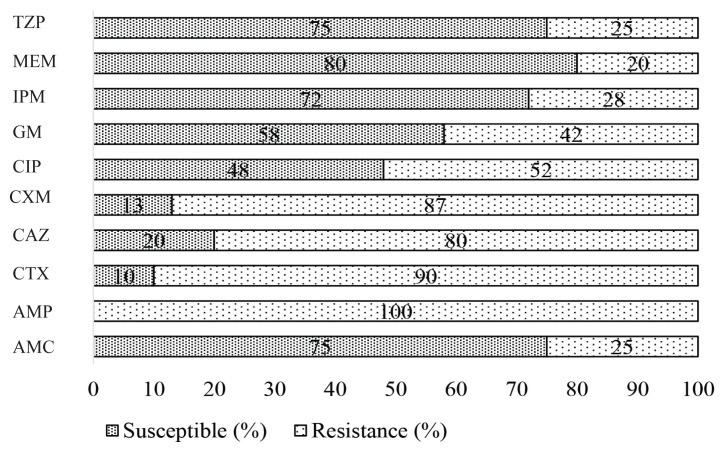
Antibiogram patterns of VTEC

The antibiogram pattern of the 29 VTEC isolates indicates that antibiotic resistance is common among most of the VTEC isolates. The results demonstrated that 90% (*n* = 26) of the isolates were resistant to CTX, followed by 87% (*n* = 25) resistant to CXM and 80% (*n* = 23) to CAZ. In addition, 100% (*n* = 29) resistant to AMP. However, the results showed that less common resistance to CIP (52%) followed by GM (42%) and IPM (28%). Moreover, all the VTEC isolates were found to be the least resistant (20%) to MEM (Figure 3). Consequently, the antibiotic susceptibility patterns of the VTEC revealed that all the isolates were significantly resistant to at least three antibiotics belonging to different classes: AMC, CEX, CTX, ATM, TZP, CAZ, CIP, MEM, IMP and FEP. Of the total, 24 (80%) strains of the VTEC were multidrug-resistant (MDR) and statistically significant (*P* < 0.001). The highest frequency of MDR isolates obtained from female patients (*n* = 16 [55%]), while 8 (27%) in males (Table 5).

AMC-30 μg, CEX-30 μg, ATM-30 μg, CIP-10 μg, CTX-30 μg, FEP-30 μg, CAZ-30 μg, IMP-10 μg, MEM-5 μg and TZP-10 μg/75 μg.

## Discussion

Diarrhoeagenic *E. coli* has become a health risk for children, particularly in developing countries (32). Various virulence factors such as verotoxin genes are attributed to non-O157 *E. coli* pathogenicity, causes illness, which ranges from mild watery diarrhoea to life-threatening complications (33). Non-O157 *E. coli* affects younger children more often compared to O157 *E. coli* (34). Also, VTEC has potential cytotoxic assays to cause severe illness that can lead to outbreaks, and these situations can transcend beyond the country’s boundary.

This present study showed that the VTEC have a relatively high potential for causing life-threatening complications such as diarrhoea and it is an agreement with several similar studies, specifically in Brazil (78.3%) (35), Canada (93.8%) (36), Iran (50%) and Malaysia (33%) (37), whereas low prevalence was reported in Iran (17.47%) (38). The pathogenic virulence properties of several VTEC serogroups, O26, O103, O111, O121, O145 and O45 serotypes were detected from 49 non-O157 *E. coli* isolates. However, these serogroups are commonly associated with severe disease outbreaks, and in some countries, are isolated from clinical samples more often than O157. In addition to the non-O157 serogroups, viz., O26, O145, O111 and O103 are more often associated with severe life-threatening complications (39). Our result showed that 8% of O26 serogroup was the most prevalent virulent factor. Similar results of O26 serogroup have been reported from Malaysia (25%), Asia-Pacific (7.2%) (40), Europe (3.5%) (41), Africa (7%) (42) and America (17%), (43) which are in agreement with our findings. However, a higher frequency of O26 serotype was observed in Iran (43.75%) (44), Malaysia (18.4%) (12) and Canada (62.6%) (45). Epidemiological surveillance indicates that the persistence of non-O157 serotypes, *E. coli* O26 is the one of the major serotypes of concern. Moreover, the O121 serotype was the second most prevalent in our findings. Studies showed that O121 had been widely associated with severe disease outbreaks (46). Additionally, serotype O26 *E. coli* caused a recent outbreaks in the USA and Mexico (47), which draws our attention as O26 *E. coli* serotype are also the most prevalent in our current findings.

Many different incidences of VTEC strains associated with severe diarrhoea. Also, virulence gene (VT2) are associated with high prevalence of VTEC and with HC or HUS (48). In the current study, the most commonly observed VTEC virulence profile included 29 (59.1%) VT1, followed by 5 (17.2%) VT2 and 3 (10.3%) strains harboured both genes (VT1 and VT2) among 49 isolates of non-O157 diarrhoeagenic *E. coli* affected children (≤ 5 years old) (Table 2). However, a recent study shows that VT2 play a significant role as a source of human infections than VT1 (49–50). Molecular detection of VT1 virulence genes is more prominent than VT2 in VTEC strains. These findings (59.1%, VT1) are reliable with a recent study performed by Shridhar et al. (51) and in contrast with Neher et al. (52) who stated that the VT2 gene was most frequent among VTEC isolates. However, VTEC isolates are associated with severe diarrhoea involving pathogenicity of VTEC virulent factors which agree with several previous studies (53–56). The high frequency of VT1 genes of VTEC strains attributes to *E. coli* infections (36). The wide variations in the prevalence of VTEC can be attributed to epidemiological determinants (57). These observations draw very significant attention viz., the prevalence of VTEC in this geographical area and these virulence genes belong to non-O157 *E. coli*. Hygienic practices, consumption of contaminated food, and consequent faecal-oral transmission make a substantial different scenario between developed and developing countries. Surprisingly, VTEC other than O157: H7 serotype is not actively reported in epidemiological settings, while non-O157 infections are increasingly recognised as significant causes of diseases, including outbreaks (5). VTEC, a substantial cause of dysentery, has also been reported in America, Europe, Asia and Africa (58–59).

Globally, microbial resistance properties among bacteria are at high risk and its susceptibility patterns depend on variation in population and environments (17, 60–61). In this present study, VTEC exhibited the highest level of resistance to AMP (100%), CTX 90%, CXM (87%) and CAZ (80%). Also, the resistance level (20% to 100%) against various classes of antibiotics was high in VTEC strains. The findings of the multidrug-resistant level (80%) were consistent with a recent study in Japan by Kusumoto et al. (62). However, 62% of the *E. coli* strains exhibited MDR in Malaysia (63). These highlights the rising trend of broad-spectrum MDR in VTEC strains. MDR strains of VTEC poses serious health hazards to human health by resisting various classes of antibiotics. Moreover, MDR interrupts or delays the treatment efficacy against it. Besides, the use of inappropriate drugs in animals and humans, and their release into the ecosystem affect antimicrobial resistance patterns. These resistant bacteria may transfer the resistance properties to other related bacterial species, which forms to multidrug-resistant strains (64). Finally, antibiotic becomes less effective, which led to infection persist in patients and increase the risk of spread worldwide.

Environmental contaminants, waste materials, non-developed sanitary and hygienic systems are a potential source of VTEC infectious pathogen prolongs illness, disability and death. However, there is no global policy statement on outbreaks control. Besides, a general overview of clinical documentation is absent on the most significant diarrhoeagenic *E. coli*, global VTEC outbreaks and diarrhoea episodes (5). The related available epidemiological information still needs to be investigated, and this will require an interactive initiative among infection control management, professionals at the clinic, public health and research level.

## Conclusion

Virulence genes and pathogenic forms of *E. coli* cause a variety of diarrheal diseases in humans, especially among children. A high frequency of VTEC serotypes associated with pediatric diarrhoea in Kuantan, Malaysia. Besides, most of the isolates were resistant to different types of antibiotics, with a higher incidence of MDR. This study suggests that health priorities could prevent VTEC strains associate diarrhoea among children. Therefore, proper hygienic practices, consumption of well-cooked food, avoid raw milk or meat and drinking recreational water could be the best preventive pathways. Also, surveillance systems monitoring need to be extended to incorporate antibiotic use, development, and dissemination of antimicrobial-resistant within clinical and ecological samples.

## Figures and Tables

**Table 1:** Primers and PCR conditions

Target gene	Primer sequences	PCR condition	Size bp	Reference
VT 1–F	5′-CAC CAG ACA ATG TAA CCG CTG-3′	94 °C for 3 min for one cycle followed by 35 cycles of 94 °C for 1 min, 65 °C for 2 min, 72 °C for 2 min and final extension 1 cycle of 72 °C for 10 min	348	(25–26, 28)
VT 1–R	5′-CAG TTA ATG TGG TGG CGA AGG-3′			
VT 2–F	5′-GCG TCA TCG TAT ACA CAG GAG C-3′		584	
VT 2–R	5′-ATC CTA TTC CCG GGA GTT TAC G-3′			

**Table 2:** Occurrence of target ‘big 6’ non-O157 serogroups from the clinical isolates of *E. coli*

Age (old months)	No. of isolates	O111	O121	O145	O26	O45	O103	*P*-value
0–12	53	1^a^	1^c^	0	0	0	1^b^	< 0.001
13–24	22	1^b^	1^a^	1^b^	3^b^	1^c^	0	
25–36	22	3^a^	3^a^	2^b^	2^a^	1^c^	1^c^	
37–48	25	2^b^	1^b^	2^c^	2^b^	3^a^	3^b^	
49–60	15	1^c^	3^a^	3^a^	4^a^	1^c^	2^a^	

Total	137	8	9	8	11	6	7	

Notes:

a Watery stools (*n* = 25 [18.2 %]);

b Mucoid stools (*n* = 16 [11.6 %]);

c Bloody stools (*n* = 8 [5.8 %])

**Table 3:** Clinical features and risk factors among children infected with diarrhoeagenic *E. coli* pathotypes

Features/risk factors		No. of isolates	Positive isolates	No. of diarrhoeagenic VTEC pathotypes			*P*-value

				VT1	VT2	VT1 and VT2	
Age (months old)	0–5	17	7	6	0	1	0.04
	6–12	36	5	3	0	2	
	13–24	22	8	6	2	0	
	25–36	22	5	4	1	0	
	37–48	25	2	1	1	0	
	49–60	15	2	1	1		
Sex	Male	71	10	5	3	2	0.03
	Female	66	19	16	2	1	
Symptoms	Vomiting	25	10	7	2	1	0.01
	Abdominal pain	32	7	4	2	1	
	Nausea	45	10	8	1	1	
	None	35	2	8	0	0	
Temperature	> 38 °C	56	18	13	3	2	0.008
	< 38 °C	81	11	8	2	1	
Feeding type	Breast milk	76	2	1	1	0	< 0.001
	Breast milk + formula milk	32	15	13	1	1	
	Solid food	29	12	7	3	2	
Diarrhoea type	Watery	84	14	9	3	2	0.05
	Mucoid	33	12	11	1	0	
	Bloody	13	3	1	1	1	
	Loose	7	0	0	0	0	
Duration of diarrhoea	1 day	22	7	5	1	1	0.04
	2–3 days	50	5	3	1	1	
	4–5 days	50	15	11	3	1	
	> 6 days	7	0	0	0	0	
	No information	8	2	2	0	0	
Admission type	Admitted	35	18	14	3	1	< 0.001
	Outpatients	90	8	5	1	2	
	No information	12	3	2	1	0	

**Table 4:** Distribution of verotoxin gene (VT1 and VT2) in non-O157 *E. coli* isolates

Virulence genes	Six serogroups of non-O157 *E. coli* (*n* = 49)						*P*-value

	O111	O121	O145	O26	O45	O103	
None	4	5	5	2	1	3	< 0.001
VT1	3	3	3	6	4	2	
VT2	1	1	0	2	0	1	
VT1 and VT2	0	0	0	1	1	1	

**Table 5:** MDR profile of VTEC isolates

Resistance	Types of antibiotics	VTEC isolates						*P*-value

		Male (*n* = 10)		Female (*n* = 19)		Total (*n* = 29)		

		No	%	No	%	No	%	
Resistance to 3 agents	IMP, TZP, CTX	4	13	9	31	13	44	< 0.001
Resistance to 4 agents	ATM, MEM, CIP, CTX	3	10	3	10	6	20	
Resistance to 5 agents	FEP, ATM, CEX, MEM, AMC	1	3	2	6	3	10	
Resistance to 6 agents	CIP, CEX, AMC, CTX, ATM, IMP	-	-	1	3	1	3	
Resistance to 10 agents	AMC, CEX, CTX, ATM, TZP, CAZ, CIP, MEM, IMP, FEP	-	-	1	3	1	3	
